# Thank you to our 2025 reviewers

**DOI:** 10.1016/j.ebiom.2026.106179

**Published:** 2026-02-11

Robert K Abbott

Carla Abdelnour

Ziad Abuhelwa

Laith Abu-Raddad

Hiroaki Adachi

Adebowale Adebiyi

Demilade Adedinsewo

Nicholas Aderinto

Louise Adermark

Cornelia Adlhoch

Joeri Aerts

Anani Afanou

Sri Mahavir Agarwal

Sean Agbor-Enoh

Rahul Aggarwal

Surya Aggarwal

Anurag Agrawal

Céline Aguer

Umberto Aguglia

Shafqat Ahmad

Sharia Ahmed

Jingwen Ai

Elmar Aigner

Diego Albani

Sebastían Albertí

Micheala Aldred

Miguel Alejandre-Alcázar

Tomas Aleman

Zorzi Alessandro

Marc Allard

Kimberly Allison

Yves Allory

Madson Almeida

Signe Altmäe

Amedeo Amedei

Nelly A Amenyogbe

Jing An

Seong An

Petronela Ancuta

Monica Andersen

Peter Munch Andersen

Douglas Anderson

Karen Anderson

Nicolas André

Matti Annala

Sabiqah Tuan Anuar

Victor Appay

Virginia Aragon

Hisashi Arase

Marcos J Arauzo-Bravo

Carla Araya-Cloutier

Nathalie Arbour

Paolo Arcidiacono

Carmen Ardanuy

Kazim Arga

Burak Arslan

Prabhu S Arunachalam

Nariaki Asada

Fotis Asimakopoulos

Francesco Asnicar

Shiu Lun Au Yeung

Arnaud Augert

Aya Awwad

Julio Ayala

Özgün Babur

Luciano Bachmann

Athanase Badolo

Vladimir P Badovinac

Sunil Badve

Ulas Bagci

Melanie Bahlo

Armita Bahrami

Lijun Bai

Yang Bai

Dalan Bailey

Sébastien Bailly

María Elvira Balcells

Frances Balkwill

Gareth Ball

Priyanka Baloni

Niaz Banaei

Abdul Rouf Banday

Anita E Bandrowski

Ritu Banerjee

Bettina Bankamp

Anju Bansal

Chang-jun Bao

Bridget Barber

Jacqueline M Barker

Shaun Barnabas

John Barnard

Esther Barreiro

Jenny Barrett

Alexander Bartelt

Alfred Bartolucci

Dinesh Barupal

Boris Bastian

Hélène Bastuji

Lisa Bauer

Michael Bauer

Thomas Baumert

Dirk Baumjohann

Robert A Beckman

Helen Bedford

Martin Beer

Andrew Beggs

Iman Beheshti

Christoph Beier

Sander Bekeschus

Eric Belin De Chantemèle

Christine Bellanné-Chantelot

Anna Bellizzi

Giovanni Bellomo

Jessica Belser

Matteo Benelli

Tamir Ben-Hur

Ariela Benigni

Mark Bennett

Felix F Benninger

Panayiotis Benos

Anna Benrick

Vivian Berg

Peter Bergman

Jose I Bernardino

Céline Berthier

Antonio Bertoletti

Joyce Besheer

Nick Betley

Pasqua Maria Betta

Martijn Beudel

Nikita Bhalerao

Balu Bhasuran

Nazim Bhimani

Nan Bi

Vasiliki Bikia

Murat Bilgel

Julie Bines

V A Binson

Matthew Birnie

Jordan E Bisanz

Aaron W Bivins

Jaishri Blakeley

Anna Blakney

Julià Blanco

Joel Blankson

Camille Blériot

Marina Bluma

Lulong Bo

Mattia Boeri

Joanne Boldison

Johannes Boltze

Joseph Bondy-Denomy

Amelie Bonnefond

Louis Bont

Jacco Boon

Peter Boor

Samudragupta Bora

Alejandra Borjabad

Soo Borson

Markus Bosmann

Elena Bossi

Marita Bosticardo

Salah Boulaaras

Izolde Bouloukaki

Vincent Bourbonne

Arnaud Bourdin

Hervé Bourhy

Bertrand Bourre

Stergios Boussios

Michelle Boyle

Steven Bradfute

Philip H Bradley

James Brašić

Susanne Breitner

Thorsten Brenner

Christopher Bretherton

Olivia Briceño

Fernando Bril

Cameron J Britton

Warwick Britton

Kaspar Broch

Garrett Brodeur

F J M Broekmans

Willem Pieter Brouwer

Christopher Brown

Jenifer Brown

Judith Bruchfeld

Timothée Bruel

Adam Brufsky

Joan S Brugge

Søren Brunak

Liam Brunham

Todd Brusko

Matthew Buas

Joseph A Buckhalt

Leo Buckley

Marcus Buggert

Jote Bulcha

Charles Burant

Kathryn Burge

Tom Burne

Fiona Burns

Felix Busch

George Butler

Joseph D Butner

Zahid Butt

Natalia Buza

Ib Christian Bygbjerg

Mario Caba

Curtis H Cai

Shirong Cai

Xiaoling Cai

Yi Cai

Dana Cairns

Sofia M Calado

Virginia Calder

Lilian Calderon-Garciduenas

Brian Callaghan

Eric Calvo

Emanuela Camera

Robert Campbell

Diego Candia-Rivera

Jason Cantera

Claudia Cantoni

Diego Cantoni

Chuanhai Cao

Hailong Cao

Kai Cao

Ke Cao

Peihua Cao

Wei Cao

Yunlong Cao

Mario Capasso

Mario Capasso

Ettore Domenico Capoluongo

Megan E Capozzi

Evie H Carchman

Anna Maria Cariboni

Owen Carmichael

Carmelo Carmona-Rivera

Amancio Carnero

Anna Caroli

Guido Carpino

E Carrillo

Rita Carsetti

Ben Carter

Calogero Caruso

Adenauer G Casali

Gregori Casals

Bonaventura Casanova-Estruch

Rosa Casas

Svenja Caspers

Ryan Cassaday

Santiago Castillo-Ramirez

Gabriella Castoria

Luigi Catacuzzeno

Daniel R Catchpoole

Katrina Cathie

Dario Cattaneo

Paolo Cavoretto

Giovanni Cazzaniga

Michele Cea

Barbara Cellini

Shan Cen

Jose Cesar Rosa Neto

Semra Çaǧlar Çetinkaya

Consuelo Cháfer-Pericás

Amit Chakrabarti

Dipshikha Chakravortty

Goylette Chami

Jason Yongsheng Chan

Karen Kar Loen Chan

Ngan Yin Chan

Kevin Brown Chandler

Tamir Chandra

Ranganathan Chandrasekaran

Hyundong Chang

Junlei Chang

Mi-Yoon Chang

Xiao Chang

Marc Chanson

Tze-Fan Chao

Phunchai Charatcharoenwitthaya

Aswin Chari

Stéphane Charpier

Siriporn Chattipakorn

Koel Chaudhury

Miguel Chavez-Fumagalli

Lianqiang Che

Alain Chebly

Peter Chedraui

Michael Chee

Chang Chen

Cheng Chen

Chung-Hwan Chen

Chun-Hong Chen

Fei Chen

Feng Chen

Gongbo Chen

Guijie Chen

Haitao Chen

Hao Chen

Haoyan Chen

Hongbin Chen

Huaiyong Chen

Jiazhou Chen

Jie Chen

Jinbo Chen

Kun Chen

Lanlan Chen

Liang Chen

Lianmin Chen

Ling Chen

Miao Chen

Miaoxin Chen

Ming-Kai Chen

Minjiang Chen

Pao-Huan Chen

Renjie Chen

Sheng Chen

Tingtao Chen

Tony Chen

Wankun Chen

Wanqing Chen

Xinchun Chen

Yahong Chen

Yong Chen

Yong-Ping Chen

Yuqin Chen

Zhitong Chen

Zhi-wei Chen

Zijiang Chen

Gong Cheng

Jason Chia-Hsien Cheng

Jian Cheng

Lei Cheng

Xiangdong Cheng

Peter Cheuka

Ching-Lung Cheung

Tek Raj Chhetri

Richeng Chian

Davide Chiasserini

Floyd Chilton

Shih-Hwa Chiou

Michael Cho

Nam-Hyuk Cho

Raymond Cho

Jung-Hye Choi

Man Ho Choi

Nicolas Chomont

Manish Choudhary

Sinjan Choudhary

Constantinos Christodoulides

Myron Christodoulides

Catherine Chu

Haiqing Chu

Hin Chu

Jianping Chu

Justin Jang Hann Chu

Shi-Feng Chu

Min-Hsiang Chuang

Anatolii Chumak

Frances Chung

Cheng Ming Chuong

Mikhail I Churnosov

Annalisa Ciabattini

Fortunato Ciardiello

Michele Ciccarelli

Frank M Cichocki

Catia Cilloniz

Philipp Cimiano

José Miguel Cisneros

David John Clancy

Jordan Clark

Marie-Annick Clavel

Cristina C Clement

Genevieve Clutton

Eleonora Cocco

Cheryl Cohen

Cemil Colak

Caroline Colijn

James Collier

Lyduine E Collij

Michael Combs

Alejandro Comellas

Luis Concha-Marambio

Nicola Conran

Vincenza Conteduca

Matthew C Cook

Jonathan D Cooper

Andrew Copas

Rob Coppes

Pierre Cordelier

Mario Cornejo-Olivas

Stephen Cose

Ahmet Coskun

François Loı¨c Cosset

Guilherme Mattos Jardim Costa

Eithne Costello

Eliecer Coto

Paul Cottu

Anna Coussens

Laura Cox

Beth Coyle

Morgan Craig

Zelieann R Craig

Jeremy Chase Crawford

Fortuno Cristina

Julio Croda

Randy Cron

Jacob Crouse

Israel Cruz

James Cuffe

Baoxia Cui

Tolga Cukur

Zoran Culig

Michael P Cummings

Thiago Cunha

Coleen Cunningham

Tony Cunningham

Lukas Cyganek

Marek Czosnyka

Ricardo da Silva Antunes

Juliano Da Silveira

Antoine Da-Costa

Hanren Dai

Lianpan Dai

Russell C Dale

Kai Dallmeier

J Damoiseaux

Andrea Danese

Ian Danilevicz

Alexander Danser

Krishna B Das

Claudia Andrea Daubenberger

Nicolas Dauby

Patrick Davis

Farshid Davoodi

Christopher J Day

Mamede de Carvalho

Emiliano de Cristofaro

Ugo De Giorgi

David de Gonzalo-Calvo

Rik De Greef

Idoia Martínez de LaPiscina

David De Oliveira

Gérard De Pouvourville

Karin de Punder

Laura P M H de Rooij

Stephen C De Rosa

Anita De Rossi

Francesco De Sanctis

Maarten De Vos

Aiko de Vries

Laurent Debarbieux

Christopher deFilippi

Marloes Dekker Nitert

Rafael Delgado

Maurizio Delvecchio

Youcai Deng

Frederik Denorme

Andriy Derkach

Thomas Desmidt

Michael Devinney

Nirav Anilkumar Dhanesha

Waljit Dhillo

Silvia Di Agostino

Silvia Di Angelantonio

Paola Di Carlo

Massimiliano Di Filippo

Francesco Di Gennaro

Rosa Di Paola

Antonio Di Sabatino

Nicoletta Di Simone

David Diamond

Don J Diamond

Lianghui Diao

Dimitri Diavatopoulos

Francisco Díaz-Corrales

Juan F Diaz-Morales

Dibson Dibe Gondim

Jean-Luc Diehl

Derk-Jan Dijk

Emi Dika

John Dillon

Theodoros Dimitroulas

Ding Ding

Ding Ding

Pingjian Ding

Yingying Ding

Zhaoping Ding

Alexander J M Dingemans

Giulio Disanto

Lisa V Doan

Akos Dobay

Prashant Dogra

Ing-Mari Dohrn

Margarita Domínguez-Villar

William Donald

Di Dong

Fajin Dong

Jingcheng Dong

Wuzi Dong

Xinran Dong

Chantal Donovan

Silvia Dossena

Florian Douam

Mark Douglas

Abigail Dove

Stuart Dowall

Kimberly Dowd

Guangwei Du

Jiangbo Du

Juan Du

Lei Du

Min Du

Xiangjun Du

Xinsong Du

Hua Duan

Raffaele Dubbioso

Mariette F Ducatez

Simon Ducharme

Emma Duerden

Rae Duncan

Timothy Q Duong

Luc Dupuis

Thomas Martin Durcan

Ralf Dürrwald

Gregory Dussor

Malcolm S Duthie

Krit Dwivedi

Paul J Dyson

Zoe Dyson

Albino Eccher

Tim Eckmanns

Eduardo Edelman Saul

Aziz Eftekhari

Camille Ehré

Anja Ehrhardt

Stéphanie Eid

Tal Einav

Avraham Eisbruch

Martin Eklund

Elie El Agha

Khaled El Emam

S A El Masry

Shokrollah Elahi

Elis Cristina Araújo Eleuthério

Kevin Elias

Michael Eller

David Elliman

Corinne Engelman

James Engert

Heather Enright

Brian Epling

Carmina Erdei

Serkan Erdin

Jan Eriksson

Roser Escrig Sarreta

Mohammed Eslam

Paolo Eusebi

D Gareth Evans

Antonio Facciorusso

Samira Fafi-Kremer

Instructor Fahrmann

Jonathan Fallowfield

Ann Falsey

Fan Fan

Ruifeng Fan

Daniele Fanale

Hai Fang

Mengjie Fang

Sonia Fargue

Mohsin Farooq

Forough Farrokhyar

Alfonso Fasano

Azadeh Feizpour

Jacques Fellay

Falko Fend

Dechun Feng

Jiexiong Feng

Ninghan Feng

Yuan Feng

Xiaoming Fengxiaoming

Joe R Fenn

Miguel Fernández García

José Manuel Fernández-Real

Erica Ferrini

Martha Feucht

Rita Fidalgo-Pereira

Jess Fiedorowicz

Nicola Filippini

Tommaso Filippini

Josef H Finsterer

Andres Finzi

Alessandro Fiorenzano

William Fischer

Valerie Flaherman

Nicola F Fletcher

Carlos Flores

Christa Flueck

Luisa Foco

Duarte Folgado

Kevin Fonseca

Francesco Fontanella

Diego A Forero

Roberta Forlano

Juan Fortea

Even Fossum

Paula J Foster

Philip Fowler

David Fox

Jennifer Frankovich

Martin Frasch

Heinz Freisling

Pim French

Mario Frias

Christoph M Friedrich

Daniel E Frigo

Giovanni Frisoni

Bernd Fritzsch

Philippe Froguel

Rémi Fromentin

Jelke J Fros

Christopher S Fry

Amy K Y Fu

Guanghui Fu

Qi Fu

Ying Fu

Yuanqing Fu

Yuhong Fu

Hans Peter Fuehrer

Jumpei Fujiki

Maki Fukami

Maria Fuller

Morgan D Fullerton

Tamas Fulop

Nicholas Funderburg

Jessica Furriol

Wakako Furuyama

Sabato Fusco

Kelsey Gabel

Martina Gabrielli

Shruti Gadewar

Lorenzo Gaetani

Carlos Gaetano

Nico Gagelmann

Daniel Gagnon

Marie Gaine

Alexander Galkin

Eleonora Galosi

Mingyu Gan

Shi-Rui Gan

Yunn-Hwen Gan

Arnold Ganser

Guofen Gao

Hai-Tao Gao

Jianli Gao

Jingwei Gao

Junjie Gao

Lifen Gao

Qian Gao

Shaorong Gao

Shugeng Gao

Tianming Gao

Xiang Gao

Yadong Gao

Antonia Garcia

Maria Garcia

Alexis García

Sonia García-Calzón

Blanca Estela García-Pérez

Paul Gastanaduy

Phillip Charles Gauger

Alban Gaultier

Martin Gauster

Surabhi Gautam

Serge Gauthier

Enrique Gayo

Diego Gazzolo

Long Ge

Heonyung Gee

Raif Geha

Jörg Geiger

Arkadiusz Gertych

Sadegh Ghaderi

Anahit Ghochikyan

Debashis Ghosh

Luca Giacomelli

Evangelos Giamarellos-Bourboulis

Kevin Gibbs

Lara Gibellini

Judy Gichoya

Bruna Gigante

Crystal M Gigante

Thomas Gillingwater

M A Gillman

Elisa Giovannetti

Vijayasree Vayalanellore Giridharan

Paul Gissen

Jennifer Glaus

Lise Gluud

Hartmut Göbel

Paul Goepfert

Ben Gold

Daniel Goldenholz

Shoshanna Goldin

Benjamin Goldstein

Diego Golombek

Maria L Golson

Maria Salomé Gomes

E Gómez-Gómez

Nardhy Gomez-Lopez

Fernando Gomollon

Qiyong Gong

Wenping Gong

Gloria Gonzalez-Aseguinolaza

Burel R Goodin

Mark Gooding

Sanne Gordijn

Ashraf S Gorgey

Andrew Gorringe

Pierre Goussard

Stephen Goutman

Elena Govorkova

Luis Graca

Christine Grady

Thomas Graillon

Emma J Grant

Stanislas Grassin-Delyle

Elin Gray

John R Greenland

Richard Greenwald

Mark S Gresnigt

Matthias Griese

Darren Karl Griffin

Joshua Grill

Andrew Grulich

Elin Grundberg

Anne Grünewald

Renata Grzela

Bing Gu

Feng Gu

Zhaohui Gu

Tianjia Guan

Wuxiang Guan

James Guarrera

Eric Guedj

Charles Guenancia

Salvador Martín Guinjoan

Huldrych Günthard

Cui Guo

Hui Guo

Huicai Guo

Jiabin Guo

Jifeng Guo

Jun-Chao Guo

Liqiong Guo

Minzhe Guo

Ting Guo

Zhigang Guo Guo

Eero Haapala

Tim Hagenacker

Omar Hahad

Juergen Hahn

William Hahn

Lukas Haider

Pranabashis Haldar

Peter J Halfmann

Mawieh A Hamad

Quirin Hammer

Dongsheng Han

Hongbin Han

Jing Han

Tianyu Han

Xiaodong Han

Allison R Hanaford

Takashi Hanakawa

Norio Hanata

Sandra Hanneman

Niels Hansen

Thomas Hansen

Tine Hansen

Torben Hansen

Chun Hao

Jie Hao

Mingang Hao

Myra Happe

Hidetaka Hara

Zitta Harboe

Joshua Hare

Michael Harhay

Tetsuhiro Harimoto

Ahmad Hariri

Arif Harmanci

Paul Harms

Violaine Harris

Luke Harrison

Jan Miroslav Hartinger

Tomonobu Hasegawa

Kenji Hashimoto

Koji Hashimoto

Jeffrey Haspel

Janna Hastings

Steen Haugaard

Daisuke Hayasaka

Guo-Wei He

Jian He

Jingni He

Peng He

Yong He

Yonghan He

Yulei He

Manfred Hecking

Haley Hedlin

Christopher Heeschen

Feng Q Hefeng

Eszter Hegyi

Ilkka Heinonen

Angela M Henricks

N Lynn Henry

Marisol Herrera-Rivero

Margaret Hibbs

Heather Highland

Hayato Hikita

Elif Hindié

Yushi Hirota

Akitoyo Hishimoto

Yuki Hitomi

Ya-Chi Ho

Berthold Hocher

Jeanne Marie Hoffman

Günter Höglinger

Boon Peng Hoh

John Bradley B Holcomb

Jens Holst

Akira Honda

Jason Hong

Kyung-Won Hong

Qingqi Hong

Shenda Hong

Jay Hooper

Frederic Hopf

Bernd Hoppe

Rita Horvath

Aniqa Tasnim Hossain

Can Hou

Dayong Hou

Likun Hou

Rein Houben

Valérie Hox

Deanne H Hryciw

Elena Wen Yuan Hsieh

Ming Ju Hsieh

Chia-Lang Hsu

Cheng Hu

Fupin Hu

Min Hu

Xiaoxia Hu

Zhihong Hu

Ziyang Hu

Jinhai Huang

Litung Huang

Min Huang

Shenglin Huang

Suli Huang

Tao Huang

Wenzhong Huang

Yu Huang

Yuanyu Huang

Zhaohui Huang

Richard Hughes

Hannah Xiaoyan Hui

Ivan F N Hung

Coen Hurkmans

Killian Hurley

William Duncan Hutchison

Tsong-Long Hwang

Per Hydbring

D S Fahmeed Hyder

Taisen Iguchi

Yasumasa Ikeda

Olusegun Johnson Ilegbusi

Irena M Ilic

Christopher Illingworth

Imre Imre Lengyel

Kentaro Inamura

Stefano Indraccolo

Carmen Iñiguez

Bruce Innis

Pasquale Innominato

Juan Carlos Irwin

Harry Ischiropoulos

Ryouhei Ishii

Kiyotake Ishikawa

Gaetano Isola

Michael Ison

Adrian Israelson

Masaoki Ito

Toshiki Iwabuchi

Katsuomi Iwakura

Marcin Iwanicki

Shigeru Iwata

Juan Carlos Izpisúa Belmonte

Zsuzsanna Izsvák

Yuishin Izumi

Susanne Jacobsson

Hartmut Jaeschke

Mathieu Jagt

Faezeh Jahedi

Sanjay Jain

Eddie James

Paul James

Mirosław Janowski

Natasha Jansz

David J Jeffries

Jeffrey Jenks

Seogsong Jeong

Katarzyna Jerzak

Niels Jessen

Hem Jha

Jia Fong Jhang

Rui Ji

Yun Ji

Deshui Jia

Wei Jia

Wei-Hua Jia

Wenwen Jia

Xuemei Jia

Zhanjun Jia

Fangyuan Jiang

Feng Jiang

Guochun Jiang

Guozhi Jiang

Haihui Jiang

Hong Jiang

Wei Jiang

Wen Jiang

Yiguo Jiang

Bin Jiao

Xinan Jiao

Xue Jiao

Zheng Jiao

Zhicheng Jiao

Joohi Jimenez-Shahed

Fengyan Jin

Kai Jin

Sheng Chih Jin

Yan Jin

Zhichao Jin

Zi-Bing Jin

Dong Hyun Jo

Dirk Jochmans

Bina Joe

Deepu John

Eric Otto Johnson

Meaghan Jones

Stipan Jonjic

Jihoon Joo

Justin Julander

Steven Julious

Hyun-Do Jung

Jaeung Jung

Wonyeong Jung

Jennifer Juno

Michael Jurczak

Kerstin Jurk

Jamie Justice

Stephen Juvet

Priyadarshini Kachroo

Tim Kacprowski

Tu'uhevaha Kaitu'u-Lino

Lorraine Kalia

Shirin Kalimuddin

Ulrich Kalinke

Thierry Kalonji Mukendi

Philip A Kalra

Rishikesan Kamaleswaran

Kenya Kamimura

Dorota Kaminska

Hannah Kaminski

Abram Kamiza

Takahisa Kanekiyo

Yonehiro Kanemura

Insoo Kang

Jeonghyun Kang

Anna Kankaanpää

Siddhartha P Kar

Panagiotis Karanis

Hassen Kared

Samuel Kariuki

Angelos Karlas

Harry Karmouty Quintana

Morten Karsdal

Yanislava Karusheva

Vivi Kasim

Belinda Kaskow

George Kassiotis

Yasufumi Katanasaka

Koichi Kato

Masahisa Katsuno

Johanna Kattenberg

Christopher Käufer

Daniel Elias Kaufmann

Anand Kawade

Yoshihiro Kawaoka

Tom Kay

Sina Kazemian

Ruian Ke

Brendan J Keating

Katherine Kedzierska

Allison M Keeler

Daniel Keeser

Maria Keller

Robert G Kelly

Mark Kelson

Zoe Kelson

Kevin Patrick Kenna

Nicholas Kennedy

Stephen Kent

Nicholas J Kenyon

Anusak Kerdsin

Attila Keresztes

Winfried Kern

Sander Kersten

Maryam Keshtkar-Jahromi

Imen Ketata

Fazlurrahman Khan

Ali Khashan

Babak Khoshnood

Mark Kidd

Fabian Kiessling

Joep Killestein

Dongin Kim

Jae Kyoung Kim

Jaeyeon Kim

James Kim

Jong Kyoung Kim

Jung Hee Kim

Kang Kim

Kenneth Kim

Ki Wook Kim

Masafumi Kitakaze

Georgios Kitsios

Alisa Kjaergaard

Robyn Klein

Kleopas A Kleopa

Vanja Klepac-Ceraj

Susanna Klevebro

Anna Klintsova

Jochen Klucken

Lori A Knackstedt

Stian Knappskog

Stuart Knechtle

Alan Paul Knutsen

Takashi Kobayashi

Daniel Koboldt

Bobby Koeleman

David M Koelle

Cristiano Köhler

Shrey Kohli

Sebastian Kohlmann

Loick Pradel Kojom Foko

Vasantha Kolachala

Yoshihiro Komohara

Bin Kong

Huihui Kong

Xiangfeng Kong

Masamitsu Kono

Michihito Kono

Konstantinos Konstantopoulos

Evangelos Kontopantelis

Irina Kontsevaya

Jill Koshiol

Kate Kosmac

Sri Rajasekhar Kothapalli

Anai Kothari

Olga Kovalchuk

Markus C Kowarik

Shohei Koyama

Julia Kozlitina

Robert Kraaij

Henry Kranzler

Susanne Krasemann

Gregory Krauss

Pamela K Kreeger

Daniëlle Krijgsman

Natasha Krishnadas

Guido Kroemer

Christopher David Kroenke

Manuel Krone

Cheng-Lung Ku

Le Kuai

Yasutoshi Kuboki

Tatiana V Kudryashova

Jens Kuhle

Louise Kuhn

Arutha Kulasinghe

Hrishikesh Kulkarni

Sanjana Kulkarni

Dileep Kumar

Kishore Kumar

Kishore R Kumar

Sudhakar Kumar

Poorbita Kundu

Wolfram S Kunz

Ho-Chang Kuo

Andreas Kupz

Om Kurmi

Takeshi Kurosu

Kenya Kusunose

V Raman Kutty

Liisa Kuula

Junghoon Kwon

Lawrence Kwong

Jean Kwun

Dohern Kym

Marco La Manna

Herbert M Lachman

Magalie Ladouceur

Oliver Laeyendecker

Dongmei Lai

Jianyu Lai

Kefang Lai

Tianwen Lai

Kerry J Laing

Nicole Lake

Igor Lakhno

Sham Lal

Santosh Lamichhane

Nathaniel Landau

Raphael Landovitz

Wolfgang Langhans

Joanne Langley

Lies Langouche

Tobias Lanz

David Largaespada

Lidia Larizza

Alicia Latham

Douglas Lauffenburger

Pierre Laurent-Puig

Carl Lavie

Elad Lax

Ahmed Lazrak

Nguyen Quoc Khanh Le

Eric Le Guern

Cyril Le Nouën

Valerie Le Sage

Benjamin Lee

Cherie Yin Lau Lee

Chih-Hsin Lee

Edward Lee

Kyung-Hun Lee

Meihsuan Lee

Min Shi Lee

Ming-Jen Lee

Seung-pyo Lee

Wanping Lee

Catalina Lee-Chang

Sophie Lefèvre-Arbogast

Noemie Lefrancq

Axel Lehrer

Suvi Katri Leivonen

Rozenn Lemaitre

Manuel Lemos

Jinghua Leng

Al Leslie

Gloria Leung

Sharon Shui Yee Leung

Michael Levin

Andrew J Levine

Michael Levraut

Yves Levy

Sharon Lewin

Candace Lewis

Morag A Lewis

Ben Li

Binkui Li

Chunyang Li

Dapeng Li

Feng Li

Hao Li

Haomin Li

Hongjun Li

Hongkai Li

Jiada Li

Jingsong Li

Junliang Li

Kangdi Li

Liang Li

Meng Li

Mengmeng Li

Ming Li

Ming Li

Mingkun Li

Mo Li

Raymond Hang Wun Li

Ruichao Li

Sheyu Li

Sung-Chou Li

Taisheng Li

Wanwan Li

Wentao Li

Xi Li

Xia Li

Xia Li

Xiaomeng Li

Xuqi Li

Yanbing Li

Yanbing Li

Yazhou Li

Yiming Li

Yuanyuan Li

Yusheng Li

Zhiqiang Li

Zhiyuan Li

Zixiao Li

Zongjin Li

Hong Liang

Jiawen Liao

Katherine Liao

Lun-De Liao

Wei-Ping Liao

M Lichterfeld

Audrie Lin

Chin Lin

Dingbo Lin

Huapeng Lin

Jue Lin

Alisa Lincke

Magnus Lindh

Maléne E Lindholm

Charlotte Ling

Daniel Lingwood

Alexander Link

Jyh-Ming Liou

Robert Lisak

Changqing Liu

Chao Liu

Chun-Yu Liu

Cong Liu

Dillys Xiaodi Liu

Feifei Liu

Gang Liu

Ganqiang Liu

Guanghui Liu

Hong Liu

Huashan Liu

Huilin Liu

Huiyu Liu

Jia Liu

Jie Liu

Jiewei Liu

Jing Liu

Jinhui Liu

Jinyao Liu

Jue Liu

Kangdong Liu

Ling Liu

Na Liu

Pengyuan Liu

Qian Liu

Qiao Liu

Ruixin Liu

Tong Liu

Xiaosheng Liu

Xing Liu

Xing Liu

Xiqiu Liu

Yan Liu

Yanhui Liu

Yaou Liu

Yong Liu

Yong-Xin Liu

Yu Liu

Yueping Liu

Yuewei Liu

Yun Liu

Yun Liu

Zheng Liu

Zhonghua Liu

Tobias Loddenkemper

Ryan W Logan

Katja Lohmann

Shee-Mei Lok

Solveig Løkhammer

Berlín L Londono-Rentería

Stephanie Longet

Per Lonning

Chung Yeng Looi

Anton Loonen

Miquéias Lopes-Pacheco

Jose Antonio Lopez-Escamez

Armando López-Guillermo

Benjamin Lopman

Nicola Ivan Lore

Van Lun Low

Matthew Lowerison

Ake Tzu Hui Lu

Binghuai Lu

Chan Lu

Cheng Lu

Chia-Feng Lu

Jiahai Lu

Jieli Lu

Juan Lu

Limin Lu

Lin Lu

Mengji Lu

Min Lu

Minjie Lu

Zhe Lu

Zhi Lu

Brandon Peter Lucke-Wold

Thomas Luft

Geanncarlo Lugo-Villarino

Grace Lui

Antonio Luna Alcalá

Christian Lunetta

David Lung

Deyi Luo

Jianwen Luo

Kui Luo

Lianxiang Luo

Sihui Luo

Tao Luo

Xiaoying Luo

Xiongjian Luo

Liem Binh Luong

Rene Lutter

Thomas Alexander Lutz

Huibin Lv

Linli Lv

Meng Lv

Min Lv

Xinyi Lv

Qifeng Lyu

Liang Ma

Xuelei Ma

Yilong Ma

Milan Macek

Arnaud Machelart

Alex E MacKenzie

Finlay Alistair Macrae

Rosalinda Madonna

Christa Maes

Frederique Magdinier

Iddo Magen

Laurent Magy

Rajani Kanta Mahapatra

Arnaud André Mailleux

Francesco Maione

Lung-Yi Mak

Kevin Maki

Ville-Petteri Mäkinen

Suchinda Malaivijitnond

Umberto Malapelle

David Malkin

Patrick Mallon

Vivianne Malmström

Helena Maltezou

Maksim Mamonkin

Effrosyni Manali

Domitilla Mandatori

Ashutosh Mangalam

Amee Manges

Reeta Mani

Maura Manion

Ferdinando Mannello

Despoina Manousaki

Mohammad Ali Mansournia

Paolo Manzoni

Daqing Mao

Fengbiao Mao

Ning Mao

Wenjun Mao

Zhengwei Mao

Peter Maple

Damiano Marastoni

Gianluca Marcaccini

Alessandro Marcello

Vincent Marconi

Gemma Marfany

Javier Marín-Morales

Richard Markham

Michael Marks

Henk Marquering

Pierre Marquet

Megan Marron

Max E R Marsden

Allison Martin

Roland Martin

Thomas R Martin

Pablo Martinez-Amezcua

Margot Martinez-Moreno

Luis Martinez-Sobrido

Lourdes Martorell

Andriy Marusyk

Atsushi Masamune

Guillemette Masse-Ranson

Angela Mastronuzzi

Lucia Mastrototaro

Sadis Matalon

Alison Mather

Cyrille Mathieu

Alexander G Mathioudakis

Alexios Matikas

Koichi Matsuda

Hiroki Matsui

Tatsuo Matsunaga

Yuki Matsushita

Pierre A Mattar

Ivan I Maximov

Chase McCann

Michael J McCarthy

John McCauley

Melissa K McConechy

Anita McElroy

Kathryn McHugh

Laura Mecozzi

Sidharth Mehan

Siddharth Mehra

Hemalkumar B Mehta

Wei Mei

Xifan Mei

Winfried Meißner

Kelly Menees

Xiaoxi Meng

Yidan Meng

Aboma Merdasa

Ilhem Messaoudi

Metodi Dimitrov Metodiev

Marije E C Meuwissen

Benjamin Meyer

Dan M Meyer

Haakon Meyer

Jeff Meyer

Martin A S Meyer

David Meyerholz

Yi Miao

Zelei Miao

Dominique S Michaud

Federica Michielin

Patrick Micke

Robert Milewski

Jeffrey S Miller

Karen Miller

Robert J H Miller

Anastasia Minervina

Xin Ming

John D Minna

Parvaneh Mirabi

Kenji Mishima

Anup Kumar Mishra

Tatsuro Misu

Eiji Miyoshi

Robert L Modlin

Joerg Moehrle

Joerg Moehrle

Catherine Moermans

Tamer M A Mohamed

Farhan Mohammad

Shekeeb S Mohammad

Meng Ling Moi

Jean-Pierre Molès

Guido Moll

Phil Molyneaux

Yukihide Momozawa

Axel Montagne

Gabriel Montaldo

Stefano Monti

Daniel Montoro

Christiane Moog

Craig S Moore

Ginny Moore

Sebastián Morales-Pison

Noel Morgan

Akimichi Morita

Sarah Morton

Filip Morys

Jonathan Mosser

Giovanni Mostile

Kenichiro Motomura

Shan Mou

Lawrence H Moulton

Fiona Moultrie

Jarrod J Mousa

Etienne Moussay

Stellah George Mpagama

Martin Mueller

Mark Muhlau

Sumit Mukherjee

Michaela C Müller-Trutwin

Benjamin Mullin

Cynthia Munier

Christian Munz

Shin-ichi Muramatsu

Shigeo Murayama

Andrew Murphy

Teemu Johannes Murtola

David Mutch

Julian Mutz

Peninah Mwenda

Jessica Mwinyi

Daniel J Myall

Elnaz Naderi

Nael Nadif Kasri

Shuichiro Nakabo

Hidewaki Nakagawa

Hidemasa Nakaminami

Ryotaro Nakamura

Tomoko Nakanishi

Ryo Nakao

Hiroshi Nakase

Hirofumi Nakatomi

Amin Nakhostin-Ansari

H Nakshatri

Abdulqadir Nashwan

Gheyath Nasrallah

Yasodha Natkunam

Kiyohisa Natsume

Eric Nauman

Chanakha K Navaratnarajah

Martijn Nawijn

Maryam Nazemipour

Mame Diarra Bousso Ndiaye

Mark Nellist

Ina Nemet

Felix Nensa

Ujjwal Neogi

Mihai Netea

Alexander Neumann

Wolf Julian Neumann

Patrick Neven

Skye Newton

Wai Yin Ng

Alan Nichol

Yu Nie

Hannakaisa Niela-Vilén

Masaaki Niino

Jo Nijs

Vladyslav Nikolayevskyy

Marko Z Nikolić

Shangwei Ning

Toshirou Nishida

Mizuho Nishio

Akira Nishiyama

Lili Niu

Johan Noble

Franklin Nobrega

Constance Noguchi

Camilla Nord

Nicola Normanno

John Norrie

Bardia Nourbakhsh

Boris Novakovic

Giuseppe Novelli

Tristin Nyman-Mallis

Adam Nyul-Toth

Helena Oakey

Antoinette O'Connor

Richard Anthony O'Connor

Patrick Oeckl

Konrad Oexle

Natalia Ogonowski

Jiwon Oh

Yohei Okada

Ioana Diana Olaru

Roberto Oleari

Morten Salling Olesen

Vesa M Olkkonen

Henrik Olsen

Elizabeth S Olson

Yosuke Omae

Pascale Ondoa

Eugene Boon Beng Ong

Zhi Yi Ong

Weg Ongkeko

Osamu Onodera

Eng Eong Ooi

Aaron Oom

Chern Ein Oon

Antonio Orlacchio

José A Ortiz

Hitoshi Oshitani

Tomiko Oskotsky

Carl Johan Ostgren

Perrie F O'Tierney-Ginn

Timothy O'Toole

Hong-Yu OU

Meriem Ouni

Qin Ouyang

Gabriel Ozorowski

Vasantha Padmanabhan

Giuseppe Paglia

Kyoungjune Pak

Paolo Palma

Carlos M Palmeira

Reinald Pamplona

Jianbo Pan

Libin Pan

Li-Long Pan

Rajanikant Panda

Nigel S Paneth

Mao Pang

Marcus Panning

Kostas Pantopoulos

Clement Papadacci

Christopher Papandreou

Sergi Papiol

Francesco Pappalardo

Dominic Paquin-Proulx

Sagar Parikh

Marc Parisien

Tae-jun Park

Tom Parks

Tony Parks

Patricia Parnet

Lucilla Parnetti

Ioannis Parodis

Nikolaos Paschalidis

Patrizio Pasqualetti

Giuseppe Passarino

Jeroen Pasterkamp

Naomi Patel

Gopal P Pathak

Arijit Patra

Srimanta Patra

Monica Patrascu

George Patrinos

Damiano Patrono

Stephane Paul

Nathella Pavan Kumar

Kowsalya Devi Pavuluri

Jacqueline Payton

Lillian Pazvakawambwa

David Pedrosa

Niels Peek

Mark Peeples

Scott D Pegan

Mariana Pehar

Zhong Pei

Andrew Pekosz

Mireia Pelegrín

Michael Peluso

Christopher Pemberton

Sarah Pendlebury

Guoping Peng

Junjie Peng

Junmin Peng

Junping Peng

Raymond B Penn

Francesca Pennati

Giorgio Pennazza

Rachel Penrod

Víctor Pérez-García

Jesus Perez-Gil

Diego Pérez-Tilve

Alex Perkins

Andras Perl

Eran Perlson

Mathieu Pernot

Ǻsa Petersén

Christopher W Peterson

Felice Petraglia

William Petri

Gavin Pettigrew

Sophie Pezet

Betty Pham

Jiang Pi

Anne Piantadosi

César Picado

Anna Picca

Ruth Pidsley

Pedro Piedra

Rembert Pieper

Alessandra Pierangeli

Denis Piérard

Brandon L Pierce

Jean-Yves Pierga

Véronique Pierrat

Marie Pigeyre

Patrizia Pignatti

Stefano Pileri

Gabriela Veronica Pinget

Lorenzo Pini

Katja Pinker

Lígia Pinto

Rosa Pinto

Liise Anne Pirofski

Marina Piscopo

Jason R Pitarresi

Punnee Pitisuttithum

Abel Plaza-Florido

Robert Podolsky

Robert Podolsky

Jerry Polesel

Justin J Pollara

Nira Pollock

Michael Pontecorvo

Michael Pontecorvo

Philip Poortmans

Kittiyod Poovorawan

Petrică C Pop

Isabel Poschke

Ewelina Poṡpiech

Bruno Pozzetto

Jyothi S Prabhu

Hridayesh Prakash

Daniel Prantner

Francesco Prattichizzo

Jochen Prehn

Johannes Preiner

Peter Preiser

Martina Prelog

Thomas Premeaux

Enrico Premi

Robin Prescott

Paolo Preziosa

Timothy J Price

Sebastian Primpke

Nicole Prince

Kurt Prins

Jerilynn Prior

Flavia Prodam

Hans Prozesky

Pilar Puente

Fabio Puglisi

Giordano Pula

Pirkko Pussinen

Jagadeesh Puvvula

Muhammad Abdul Qayyum

Hongbo Qi

Qibin Qi

Shouliang Qi

Xingshun Qi

Kun Qian

Jin Wei Qiang

Liang Qiao

Xianhui Qin

Yun Qiu

Jie-ming Qu

Shaogang Qu

Giorgio Quer

Alfredo Quinones-Hinojosa

Richard Quinton

Ravi Radhakrishnan

Haloom Rafehi

Pawan Raghav

Paolo Ragonese

Diego Raimondo

Rupesh Raina

Taneli Raivio

Rahul Raj

Kuldeep Rajpoot

Anup Ramachandran

Nirupama Ramadas

Marie Anne Rameix-Welti

Heily Ramirez

Julia Ramirez

Alberto Javier Ramos

Robert Ramsay

Parmjeet S Randhawa

Gauri Rao

Lang Rao

Rino Rappuoli

Karen Raraigh

Sherif Rashad

Andrea Rasola

Maryam Rassouli

Philip N Rather

Alexander Rau

Cassandra Rauert

Emanuele Rausa

Nicholas M Ravanelli

Christopher Rayner

Ronny Redlich

Keith Reeves

Guislaine Refrégier

David Rehkopf

Elisabeth Reid

Jianan Ren

Siqiang Ren

C N Rensen

Dorene M Rentz

Rodolfo Rey

Rodolfo Rey

Anny Reyes

Luis Reyes

Samuel Rhedin

Hyungjin Rhee

Joon Haeng Rhee

Marcus Richards

Jennifer K Richer

Bradley Richmond

Robert Rieben

Garcilaso Riesco-Eizaguirre

Luisella Righi

Sebastian A Riquelme

Mariona Rius

Nilo Riva

Alberto Rizzo

Flavio Rizzolio

Merlin Robb

John Robbins

Olivier Robineau

Leanne Robinson

Maria Sheila Guimarães Rocha

Russell Rockne

Michael Roden

Leonardo Roever

Sheryle R K Rogerson

Hyun Cheol Roh

Simona Rolla

Miguel Romero-Perez

Katharina Ronacher

Panying Rong

Zhen Rong

Carlo Ronsini

G Ronzitti

Alessandro Rosa

Bruce Rosa

Laura Rosanò

Joshua Rosenblat

Felix Rosenow

Ted Ross

Yaron Rotman

Martin Rottman

Jean-Pierre Routy

Christopher Rowe

Louise Rowntree

Krishnendu Roy

Nitai Roy

Paula Rozenfeld

Christopher Rudd

Assaf Rudich

Agustín Ruiz Laza

Ezequiel Ruiz-Mateos

Jørgen A Rungby

Thomas Russo

Rachel Rutishauser

Feargal Ryan

Mart Saarma

Wadad Saba

Joseph Sabatino

Caroline Sabin

Suresh Anand Sadananthan

Saud Sadiq

Xavier Saelens

Ram Sagar

Narjes Saheb Sharif-Askari

Mustafa Sahin

Debashis Sahoo

Yauba Saidu

Yasushi Sakata

Hiroshi Sakaue

John Salamone

Gonzalo Salazar de Pablo

Samira Salihovic

Mirella Salvatore

Steven Salzberg

Maurilio Sampaolesi

Gloria Sanchez

L Nelson Sanchez-Pinto

Johan Sandberg

Charles Sande

Nicholas Stephen Rennie Sanderson

Soichi Sano

Brian E Sansbury

Luis Francisco Santamaría-Babí

Nayantara Santhi

Pierre Santucci

Ancor Sanz-García

Elisabetta Sarasso

Hikori Sasaguri

Kazunari Sasaki

Masanori Sasaki

M Sassani

Santosh Kumar Satapathy

David Savage

Diego Sbardella

Jakub Scaber

Georgia Schafer

Alexander Scheffold

Simona Scheggi

Edward Schenck

Francesco Schettini

Joshua Schiffer

Leonhard Schilbach

Johannes Carolus Magnus Schlachetzki

Zachary J Schlader

Andrea A Schlegel

Leopold F Schmetterer

Carsten Schmidt-Weber

Daniel Schneditz

Stefan Schneeberger

Bernard Laurent Schneider

Carolin Victoria Schneider

Ruth Schneider

Kristian Schønning

C Schooling

David Schrama

Ralf Schubert

Lara S U Schwab

Nicholas Schwab

Sebastian Schwaminger

David Schwartz

Nicola Scichilone

Giorgio Scivoletto

Robert Scofield

Daniel Scott-Algara

Kelly Seaton

Sylvain P Sébert

Sanaz Sedaghat

Anna Seekatz

Neha Sehgal

Rafick-Pierre Sékaly

Johann Sellner

Jean-Paul Selten

Axel Seltsam

John Semple

C J Seneviratne

Utpal Sengupta

Nuno Sepúlveda

Francesco Sera

Martina Sester

Aishwarya Seth

Abeer Shaaban

Baoci Shan

Chao Shan

Jinjun Shan

Manu Shankar-Hari

Denton Shanks

Kathleen M Shannon

Anantharaman Shantaraman

Yiming Shao

Yingkuan Shao

Jaqueline Shaw

Yunlang She

Gregory C Shearer

Yvette Sheline

Jana Shen

Junjie Shen

Mingwang Shen

Nan Shen

Qian Shen

Yongchun Shen

Zaki Sherif

Qinghua Shi

Meghan H Shilts

Masayuki Shimada

Kaoruko Shimizu

Hideki Shimomura

Chaewon Shin

Sung Jae Shin

YiRang Shin

Divya Shinde

Ken Shirato

Igor Shmarakov

Yehuda Shoenfeld

Sujan Shresta

Gautam Shroff

Chang Shu

Ni Shu

Xiang Shu

Xintao Shuai

Sandy Shultz

Cathy Shyr

Fusheng Si

Lu Si

Jason Sicklick

Waldemar Siemens

Elwira Sieniawska

Paul A Sieving

Alberto Signore

Nanna Sijtsema

Robert F Siliciano

Herman Silljé

Viviana Simon

Cassandra A Simonich

Abhishek Kumar Singh

Yogesh Singh

Harald H Sitte

Parco Siu

Pradeesh Sivapalan

Folke B Sjöberg

Karolina Skonieczna-Żydecka

David Skurnik

Mikael Skurnik

Riemer Hja J A Slart

Mark A Slevin

Roderick Slieker

Kwinten H E W J Sliepen

Piotr Slomka

Benjamin Smarr

David Smith

Sander Smits

Joost Smolders

Igor Smolenov

Nicolas Roydon Smoll

Hon-Cheong So

Daniel E Soffer

Mehmet Sofuoglu

Luisa Solís Soto

Daichi Sone

Bin Song

Jia Song

Juquan Song

Pengfei Song

Qianqian Song

Steven Soper

Kjetil Soreide

Christian Sorg

Alex Soriano

Carles Soriano-Mas

Vera Sorin

Adrian Soto-Mota

H Peter Soyer

Daniel J Spakowicz

Alberto Spalice

John Spencer

Jessica R Spengler

Ashley St. John

Gregg D Stanwood

Anna Starshinova

Tobias Stauber

Kathy Steece-Collier

Norbert Stefan

Sven Marcel Stefan

Katja Steiger

Eike Steinmann

Ina Annelies Stelzer

Stefan Stender

Ulrik Stervbo

Frederik Steyn

Karin Stiasny

Marie-Pierre St-Onge

Drozdstoy Stoyanov

Tomas Strandin

Sabrina Strano

Sabine Strehl

Rob T Striker

Erich Sturgis

Bin Su

Chang Su

Lishan Su

Matthew Su

Zheng Su

Marc Suárez-Calvet

Carmen C Sucharov

Stefan Suciu

Motoyuki Sugai

Makoto Sugaya

Masahiro Sugimoto

David Sullivan

Chi Sun

Dianjianyi Sun

Hong Sun

Huiyong Sun

Jianfei Sun

Jing Sun

Jing Sun

Lin Sun

Lingyun Sun

Liu Sun

Pengfei Sun

Wei Sun

Weina Sun

Xiaomin Sun

Yun Sun

Zhihong Sun

Sankaran Sundaresan

Hoon-Ki Sung

Jung-joon Sung

Pil Soo Sung

Shengfeng Sung

Rahul Suryawanshi

Melissa A Suter

Yoichi Sutoh

Naoki Suzuki

Tadaki Suzuki

J Peter Svensson

Walter Swardfager

Russell Swerdlow

William Edward Swords

Vahidullah Taç

Raquel Taddei

Yu Tahara

Usman A Tahir

Phil Tai

Ryousuke Takahashi

Kazuo Takayama

Hiroyoshi Takeuchi

Daiki Takewaki

Howard Takiff

David Talavera

Anthony Tan

Chee Wah Tan

Jen Hong Tan

Kelvin Bryan Tan

Nguan Soon Tan

Tricia Tan

Wenjie Tan

Youran Tan

Nobuyuki Tanaka

Beisha Tang

Hailin Tang

Julian Tang

Man-Hung Tang

Patrick Ming-Kuen Tang

Ruqi Tang

Shao-tao Tang

Weiming Tang

Xiangdong Tang

Yamei Tang

Qing-Qing Tao

Sheng-Ce Tao

Shi-Cong Tao

Stefano Tarantini

Andrei I Tarasov

Adi Tarca

Philip Tarr

Chandra Tayade

Angela Taylor

Hugh Taylor

Salvatore Tedesco

Andrew F Teich

Rakesh Tekade

Matthias Tenbusch

Felipe Ten-Caten

Edmond Teng

Lisong Teng

Rujeng Teng

Weiping Teng

Jeremy Teoh

Gisela Terwindt

Andrew E Teschendorff

Yihenew Tesfaye

Nandor Than

Velmurugan Thavasi

Sébastien Thériault

Isabelle Thiffault

Chloe L Thio

Kevin V Thomas

Molly Thomas

Nicholas Thomas

Mads Thomassen

Morgan Thomsen

Hans Hermann Thulke

Guo-Bao Tian

Qu Tian

Weidong Tian

Yu Tian

Yu Tian

Pentti Tienari

John Mick Tilford

Jean-Francois Timsit

Ingeborg Tinhofer

Gizachew Tiruneh

Stephen H D Tisch

Tinatin T Tkemaladze

Kelvin To

Shusuke Toden

Martin Tolstrup

Jeffrey Tomalka

Aiping Tong

Sheng Tong

Thierry Tordjmann

Mary Torregrossa

Taoka Toshiaki

Takumi Toya

Hidenori Toyoda

Gerdien Tramper

Lydie Trautmann

Mark Travassos

Jonel Trebicka

John Tregoning

Andreas Triantafyllopoulos

Joanne Trinh

Alexandra Trkola

Jakob Troppmair

Marius Trøseid

François Trottein

Leendert Adrianus Trouw

Dean Troyer

Miles Trupp

Hubert M Tse

Hung-Fat Tse

William Ka Fai Tse

Igor Tsigelny

Hiroyuki Tsuchiya

Shinobu Tsuzuki

Toyonori Tsuzuki

Edouard Tuaillon

Gro Tunheim

Qingzhang Tuo

Jacquelyn Turcinovic

Antonio Tursi

Stuart Turville

Jaakko S Tyrmi

Yuto Uchida

Daiju Ueda

Toshihiro Uesaka

Hironobu Umakoshi

Simon Urschel

Allan Vaag

Elizabeth Vafiadaki

Daniel Vaiman

Seppo Vainio

Ana Paula C Valente

Rodrigo Wladimir Valenzuela Báez

Nathalie Van Den Berge

Johanna van den Hout

Michiel van der Flier

Renate van der Molen

Stefan van Duijvenboden

Michael van Es

S van Norden

Koen Van Rompay

David Van Wagoner

Bart Vanaudenaerde

Thibaut Vanbaelen

Ilse Vanhorebeek

Eugeen Vanmechelen

Julian Varghese

Martine Vaxillaire

Juha Väyrynen

Maria Vehreschild

Sithembiso Velaphi

Raman Velayudhan

Philip Verhoef

Saguna Verma

Victor M Victor

Didac Vidal-Pineiro

Vincent Vieillard

Solveig Vieluf

Alessia Vignoli

Eduardo Vilar

Alma Villaseñor

Carlo Viscomi

Leo Visser

Rui Vitorino

Thomas Vogl

Mitchell von Itzstein

Max von Kleist

Thomas von Zglinicki

Barbara Vona

Agathe Vrillon

Girija Narendrakumar Wagh

Angelika Wagner

Matias Wagner

Hiroaki Wakimoto

Christopher S Walker

Roddy Walsh

Stephen Walsh

Thierry Walzer

Baihan Wang

Changsong Wang

Chao Wang

Chengdi Wang

Congrong Wang

Congzhou Wang

Dao Wen Wang

Dong Wang

Duolao Wang

Fangfang Wang

Fu-Sheng Wang

Haibo Wang

Hailong Wang

Hua Wang

Hui Wang

Hui Wang

Jian Wang

Jianfeng Wang

Jianjun Wang

Jianxin Wang

Jiasi Wang

Jie Wang

Jin Wang

Julia Wang

Kai Wang

Kang Wang

Kasper S Wang

Lihua Wang

Mengyu Wang

Minghuan Wang

Nan Wang

Pei Wang

Peng Yuan Wang

Qiang Wang

Qiao Wang

Qiling Wang

Qing Wang

Qingbo S Wang

Ran Wang

Rong Wang

Shu-Kui Wang

Tao Wang

Tao Wang

Tianyun Wang

Ting Wang

Wei Wang

Wei Wang

Xianwen Wang

Xiao Wang

Xiaojie Wang

Xin Wang

Xuefeng Wang

Yajuan Wang

Yang Wang

Yaqing Wang

Yeming Wang

Yi Wang

Yi Wang

Yibin Wang

Yinu Wang

Yong Wang

Youchun Wang

Zhang Wang

Zhen Wang

Zhenglu Wang

Zhengting Wang

Zhijie Wang

Zhong Wang

Celestine Wanjalla

Fredrik Wärnberg

Blake Warner

Genoa R Warner

Akira Watanabe

Satoru Watanabe

Ralf Watzlawick

Richard J Webby

Bonnie L Webster

James Weger-Lucarell

Grzegorz Wegrzyn

Chase Wehrle

Dongqing Wei

Hongjiang Wei

Hui Wei

Wei Wei

Norbert Weidner

Alexander Weigard

Adriana Weinberg

Howard Weiner

Sebastian Weis

Serge Weis

Jeffrey Neal Weiser

Guenter Weiss

Eva Weissinger

Timothy Wells

Fuqiang Wen

Zhenke Wen

Sally Wenzel

Andrew Phillip West

John T West

Lori West

Henk-Jan Westeneng

Francisco Westermeier

Nicolai Wewer Albrechtsen

Patrick Weydt

Adam Wheatley

Fletcher White

Melanie White

Nicola White

Robyn N Whitney

Emerson Wickwire

Aurelie Wiedemann

Kevin Wiehe

Alex Wiesman

Jeremy Wilkinson

Katalin Wilkinson

Daryl Lindsay Williams

Kenneth Williams

Mark Wills

Douglas Wilson

Lindsay Wilson

Eytan Wine

Y K Wing

Beate Winner

Bettina Winzeler

Slawomir Wolczynski

Roman Wölfel

Daniel Wolff

Isabelle Wolowczuk

Nicole Wolter

Grace Wong

Jason Wong

Joshua K Wong

Ngai Sze Wong

Sunny Wong

Sunny Y Wong

Neil Wright

Benjamin Wu

Chang-fu Wu

Gregory Wu

Haotian Wu

Huan Wu

Hui Wu

Jianjun Wu

Jing Wu

Lili Wu

Nan Wu

Qi-Jun Wu

Qingqing Wu

Shanshan Wu

Shaowei Wu

Song Wu

Xifeng Wu

Yao Wu

Yonghui Wu

Gerburg Wulf

Mark Wunderlich

Alexander Wyatt

Wang Xi

Zhengxiong Xi

Daniel Xia

Min Xia

Ningshao Xia

Weiming Xia

Yankai Xia

Man Xiao

Mingbing Xiao

Xinhua Xiao

Yi Xiao

Biao Xie

Dong Xie

Guoxiang Xie

Jindong Xie

Jun Xie

Lixin Xie

Xin-hui Xie

Yuntao Xie

Wenyu Xing

Xiaofang Xing

Wei Xiong

Chuanlai Xu

Gaosi Xu

Jinbin Xu

Jin-Fu Xu

Kailiang Xu

Lingling Xu

Min Xu

Ren Xu

Rongbin Xu

Shili Xu

Suowen Xu

Xiaoding Xu

Yang Xu

Ying-Chun Xu

Zhiwei Xu

Zongli Xu

Jianxin Xue

Liang Xue

Lixiang Xue

Wei Xue

Zijun Xu-Monette

Ichiro Yabe

Dharmendra Kumar Yadav

Pragya Yadav

Kentaro Yamada

Yasuhide Yamada

Shinya Yamamoto

Tatsuya Yamamoto

Ayumu Yamashita

Seiya Yamayoshi

Chao Yan

Dongsheng Yan

Hao Yan

Jian Yan

Yaping Yan

Bin Yang

Bo-Yi Yang

Chun Yang

Dawei Yang

Guangrui Yang

Guodong Yang

Hailong Yang

Ian Yang

Jun Yang

Kai Yang

Kun Yang

Liang Yang

Lili Yang

Mia Yang

Minghua Yang

Pengfei Yang

Ping Yang

Qingling Yang

Qiongqiong Yang

Shiming Yang

Tielin Yang

Ting Yang

Weihua Yang

Xinglong Yang

Xueli Yang

Yi Yang

Zongcheng Yang

Ziv Yaniv

Guidong Yao

Paul Yao

Shaohua Yao

Song Yao

Xiaojun Yao

Xudong Yao

Anat Yaskolka Meir

Rika Yasuhara

Melinda Yates

Duygu Yazici

Kaixiong Ye

Shuang Ye

Tinghong Ye

Youqiong Ye

William Shu Biu Yeung

Hyonseung Yi

Kun Yin

Liangying Yin

Shankai Yin

Wanhong Yin

Jianming Ying

Terry Yip

Song Yongmei

Soonhyun Yook

Yusuke Yoshioka

Chenyu You

Seng Chan You

Jessica Elaine Young

Chenglong Yu

Chuanjin Yu

Dianke Yu

Haitao Yu

Jianchun Yu

Jinhua Yu

Jin-Tai Yu

Lei Yu

Ling Yu

Yunsong Yu

Zuoren Yu

Chen Yuan

Lijun Yuan

Shuai Yuan

Shuofeng Yuan

Yixuan Yuan

Yong Yuan

Yongyi Yuan

Min Yue

Hongwa Yung

Simona Zaami

Marco Zampoli

Elodie Zana-Taïeb

Ladan Zand

Deirdre L Zander-Fox

Gian Franco Zannoni

Ali Zarrinpar

Michael Zech

Yashar Zeighami

Dong Zeng

Ke Zeng

Peter Zeng

Ping Zeng

Xiao-Wen Zeng

Yanni Zeng

Lina Zgaga

Qixiao Zhai

Hanxiang Zhan

Peng Zhan

Xiaowei Zhan

Anna Zhang

Bin Zhang

Bo Zhang

Bo Zhang

Chao Zhang

Chencheng Zhang

Faming Zhang

Fan Zhang

Fengqing Zhang

Fuquan Zhang

Guochao Zhang

Guochun Zhang

Guoliang Zhang

Heng Zhang

Jing Zhang

Jingren Zhang

Jun Zhang

Jun Zhang

Junxia Zhang

Lingling Zhang

Lingmin Zhang

Liwen Zhang

Liyang Zhang

Peng Zhang

Ping Zhang

Qi Zhang

Qing Zhang

Rong Zhang

Shu Zhang

Shuyang Zhang

Tianlong Zhang

Tiejun Zhang

Weimin Zhang

Xiang Zhang

Xiangyu Zhang

Xiangzhou Zhang

Xiaodong Zhang

Xin Zhang

Xin Zhang

Xuan Zhang

Yan Zhang

Yan Zhang

Yanqiao Zhang

Yi Zhang

Yonggang Zhang

Youqian Zhang

Yu Zhang

Yudong Zhang

Yunhui Zhang

Zhaocai Zhang

Zhongheng Zhang

Fengmin Zhao

Guoguang Zhao

Guohua Zhao

Huiying Zhao

Junxing Zhao

Kai Zhao

Lei Zhao

Ling Zhao

Qiang Zhao

Qin Zhao

Sen Zhao

Shidou Zhao

XiangYu Zhao

Xiaoyu Zhao

Xinyan Zhao

Yue Zhao

Zijie Zhao

Hongyi Zhào

Guohua Zhen

Beiwen Zheng

Guangrong Zheng

Ju-Sheng Zheng

Ming Zheng

Shan Zheng

Xiaobin Zheng

Yuanyi Zheng

Fangmin Zhong

Jiu-Chang Zhong

Sheng Zhong

Wenzhao Zhong

Bin Zhou

Hua Zhou

Jiancang Zhou

Jiandong Zhou

Meng Zhou

Qing Zhou

Qingjun Zhou

Wenhao Zhou

Xiangyu Zhou

Xiaoming Zhou

Xuezhong Zhou

Xu-jie Zhou

Yiming Zhou

Zhijun Zhou

Benpeng Zhu

Changlian Zhu

Chen Zhu

Haiyan Zhu

Hongtu Zhu

Jiajia Zhu

Jingjing Zhu

Lixin Zhu

Meng Zhu

Ruixin Zhu

Wusheng Zhu

Xueqiong Zhu

Yanmei Zhu

Yao Zhu

Zezhang Zhu

Stefano Ziccardi

Jakub Zmajkovic

Yutian Zou

Zhiyong Zou

Xiongbing Zu

Zachary Zumsteg

Xi-Nian Zuo

